# The Great Pretender in the Sella: A Rare Doughnut-Shaped Partially Thrombosed Internal Carotid Artery Aneurysm Mimicking Pituitary Macroadenoma

**DOI:** 10.7759/cureus.106865

**Published:** 2026-04-11

**Authors:** Ijan Dhamala, Subash Phuyal, Shivali Rao, Sashi Tandan, Gopal Sedain

**Affiliations:** 1 Neurosurgery, Tribhuvan University Teaching Hospital (TUTH), Kathmandu, NPL; 2 Neuroimaging and Interventional Neuroradiology, Tribhuvan University Teaching Hospital (TUTH), Kathmandu, NPL

**Keywords:** doughnut-shaped aneurysm, endovascular treatment, internal carotid artery, partially thrombosed aneurysm, supraclinoid segment

## Abstract

Sellar and suprasellar lesions are usually attributed to pituitary adenomas, craniopharyngiomas, and other nonvascular pathologies. Rarely, however, a partially thrombosed intracranial aneurysm can present as a tumor-like mass and create a dangerous diagnostic trap. We report a 53-year-old woman who presented with multiple episodes of vomiting for three days and intermittent headache. She had no history of loss of consciousness, seizure-like activity, or trauma, and her examination was neurologically intact, including normal visual acuity. Magnetic resonance imaging performed at another hospital showed an enlarged sella containing a 15.8 × 17 × 16 mm sellar-suprasellar lesion with a peripheral low-signal rim, central isointensity, marked T2-weighted hypointensity, and rim enhancement, leading to an initial impression of pituitary macroadenoma or craniopharyngioma. Further vascular imaging demonstrated a medially directed aneurysmal outpouching from the supraclinoid segment of the right internal carotid artery with central thrombus, producing the classic doughnut-shaped appearance. The patient underwent successful endovascular flow-diverter placement through a right femoral approach with excellent wall apposition and marked contrast stasis within the aneurysm sac. This case emphasizes that atypical sellar lesions, especially those showing T2-weighted hypointensity, hyperdensity on computed tomography, and a peripheral enhancement pattern, require vascular evaluation before any surgical plan is made. Early recognition prevents catastrophic hemorrhage and allows minimally invasive endovascular treatment with excellent anatomical results. The present case is clinically instructive because the lesion was small enough to resemble a routine sellar mass, yet dangerous enough to alter management completely once its vascular origin was recognized.

## Introduction

The sellar and suprasellar compartments contain a broad differential diagnosis that commonly includes pituitary adenoma, craniopharyngioma, Rathke cleft cyst, meningioma, inflammatory lesions, and, less commonly, vascular abnormalities. Because pituitary adenoma remains the most frequent diagnosis in daily practice, uncommon vascular lesions may be overlooked when they simulate a solid or cystic tumor. Reviews of sellar pathologies that mimic pituitary tumors emphasize that aneurysms remain among the most dangerous masqueraders because mistaken biopsy or transsphenoidal surgery can lead to catastrophic bleeding [1].

Partially thrombosed aneurysms are particularly deceptive because intraluminal thrombus alters their signal characteristics and may hide the vascular lumen on routine magnetic resonance imaging. Tamaki and colleagues described the “donut-shaped” aneurysm as a rare subtype of partially thrombosed aneurysm characterized by a patent enhancing peripheral channel surrounding thrombus, while a recent case series confirmed that these lesions remain uncommon and diagnostically challenging even in experienced cerebrovascular centers [2,3]. In parallel, flow-diverter technology has expanded the therapeutic options for complex internal carotid artery aneurysms and has been successfully adopted in resource-limited settings [4]. Misdiagnosis of such lesions can lead to catastrophic hemorrhage if treated surgically. Partially thrombosed aneurysms in the sellar region are rare, making diagnosis challenging and emphasizing the need for careful evaluation [1]. We report a rare doughnut-shaped partially thrombosed aneurysm of the supraclinoid segment of the right internal carotid artery that closely mimicked a pituitary macroadenoma on initial imaging.

## Case presentation

A 53-year-old woman presented to the emergency department with recurrent vomiting and intermittent headache. The vomiting had occurred in multiple episodes over three days. She denied loss of consciousness, abnormal body movements, recent trauma, fever, neck stiffness, visual loss, or focal weakness. She was a known hypertensive on regular medication. On arrival, she was alert, oriented, and hemodynamically stable. Her Glasgow Coma Scale score was 15/15. Both pupils were equal and reactive to light. Motor examination showed full power in all four limbs, sensory examination was normal, and no cerebellar or meningeal signs were present. Visual acuity was 6/6 in both eyes, with no clinically apparent visual field deficit. No abnormalities were detected on systemic examination. Laboratory investigations were within normal levels (Table 1).

**Table 1:** Laboratory values of the case. ESR: erythrocyte sedimentation rate

Investigation	Observation result	Reference range
Hemoglobin	14.5 g/dL	13.0-17.0 g/dL
Platelet count	238,000/mm^3^	150,000-450,000/mm^3^
Total leukocyte count	8,800/mm^3^	4,000-11,000/mm^3^
Prothrombin time (PT)	11.5 seconds	11-13.5 seconds
International normalized ratio (INR)	1.0	0.9-1.2
Activated partial thromboplastin time (aPTT)	32.0 seconds	25-35 seconds
Serum creatinine	0.9 mg/dL	0.6-1.3 mg/dL
ESR	16 mm/hr	0-20 mm/hr
C-reactive protein (CRP)	3.0 mg/L	<5 mg/L

**Figure 1:**
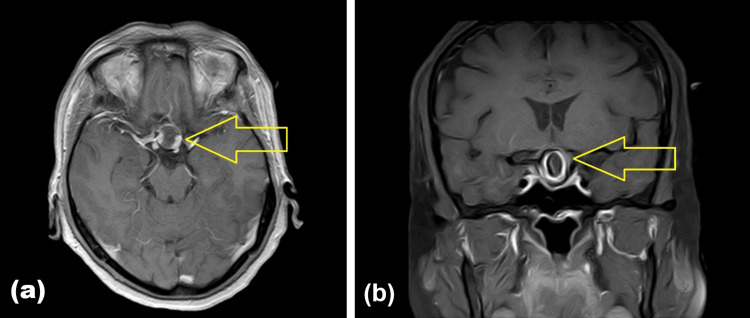
Magnetic resonance imaging (MRI) of the brain. (A) Axial T1-weighted post-contrast image demonstrating a sellar-suprasellar lesion with peripheral rim enhancement and a relatively hypointense central component (arrow). (B) Coronal T1-weighted post-contrast image demonstrating a doughnut-shaped lesion with suprasellar extension, pituitary stalk effacement, and abutment of the optic chiasm (arrow).

Magnetic resonance imaging of the brain performed at an outside hospital before presentation showed an enlarged sella. Within the sellar and suprasellar region, there was a lesion measuring approximately 15.8 mm in transverse dimension, 17 mm in craniocaudal dimension, and 16 mm in anteroposterior dimension. The lesion showed a peripheral rim of low signal intensity on T1-weighted sequences with a relatively isointense center. It was predominantly low signal on T2-weighted images and demonstrated rim enhancement after contrast administration. There was no evidence of restricted diffusion, hemorrhage, or fluid-fluid level. The lesion extended superiorly into the suprasellar cistern, effaced the pituitary stalk, and abutted the optic chiasm. It also abutted the bilateral internal carotid arteries. A thin rim of enhancing pituitary tissue was seen along the floor of the sella, and the normal intrinsic T1 hyperintensity of the posterior pituitary lobe was preserved (Figure 1). Based on these findings, the outside impression favored pituitary macroadenoma, with craniopharyngioma considered as a differential diagnosis.

**Figure 2:**
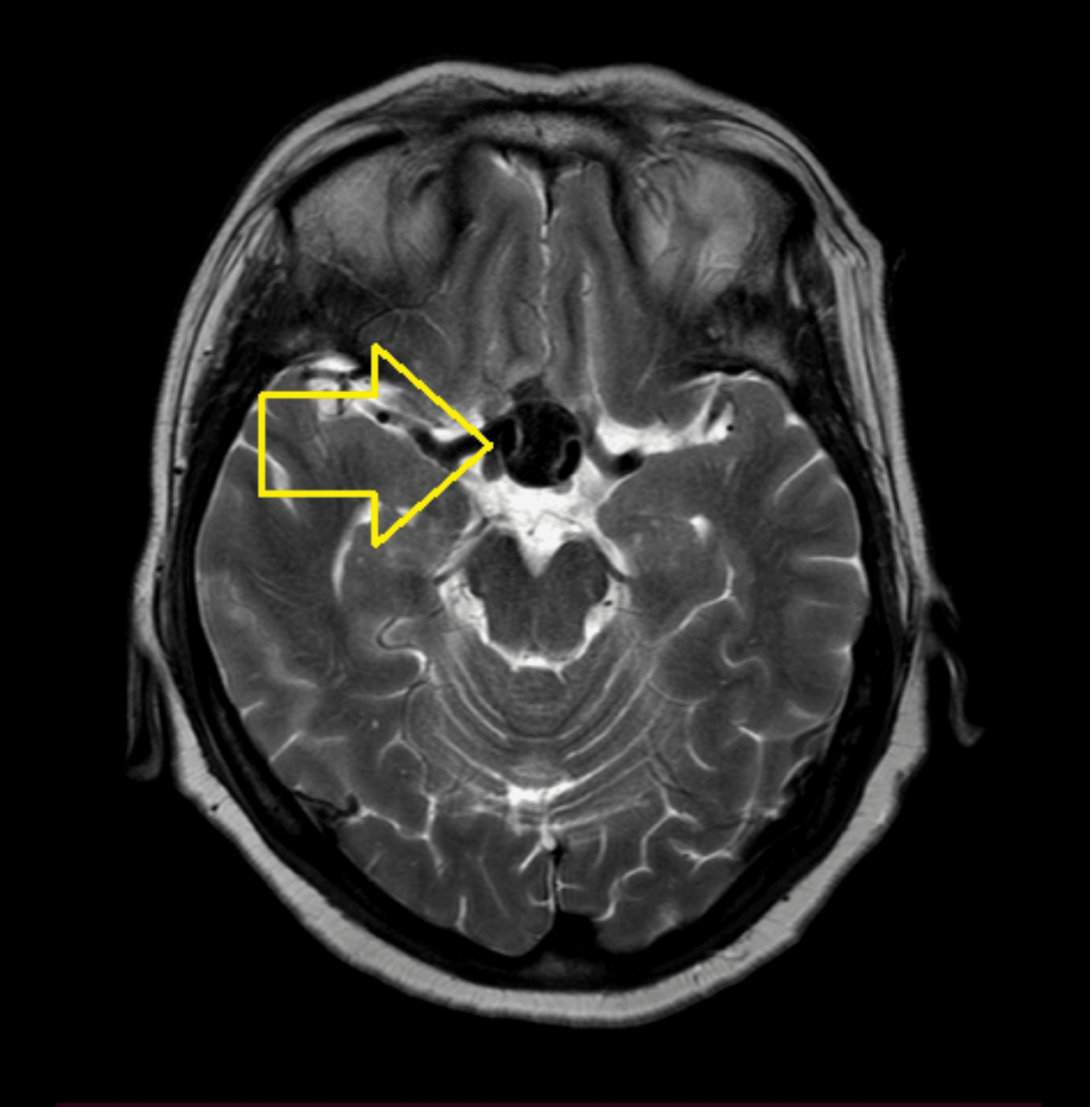
Axial T2-weighted MRI showing a well-defined suprasellar lesion demonstrating marked hypointensity, in close proximity to the internal carotid arteries (arrow). MRI: magnetic resonance imaging

Because the lesion showed striking T2-weighted hypointensity (Figure 2), close contact with the internal carotid arteries, and a peripheral enhancement pattern, further vascular evaluation was performed.

**Figure 3:**
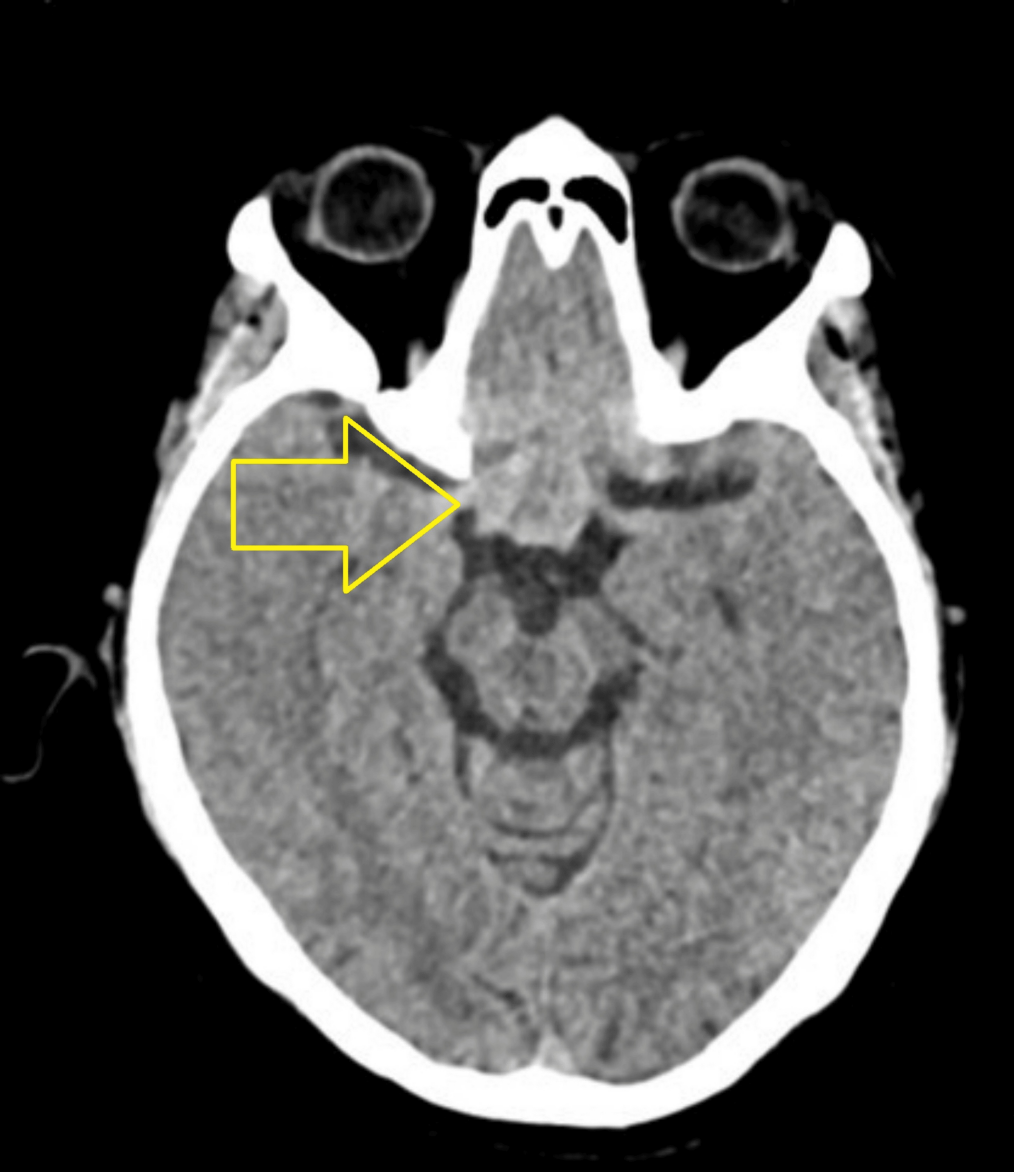
Non-contrast CT of the brain (axial image) showing a hyperdense suprasellar lesion (~65 Hounsfield units), suggestive of a thrombosed vascular lesion (arrow). CT: computed tomography

A plain computed tomography scan performed upon the patient’s presentation to our center revealed a hyperdense suprasellar mass-like lesion measuring approximately 65 Hounsfield units (Figure 3).

**Figure 4:**
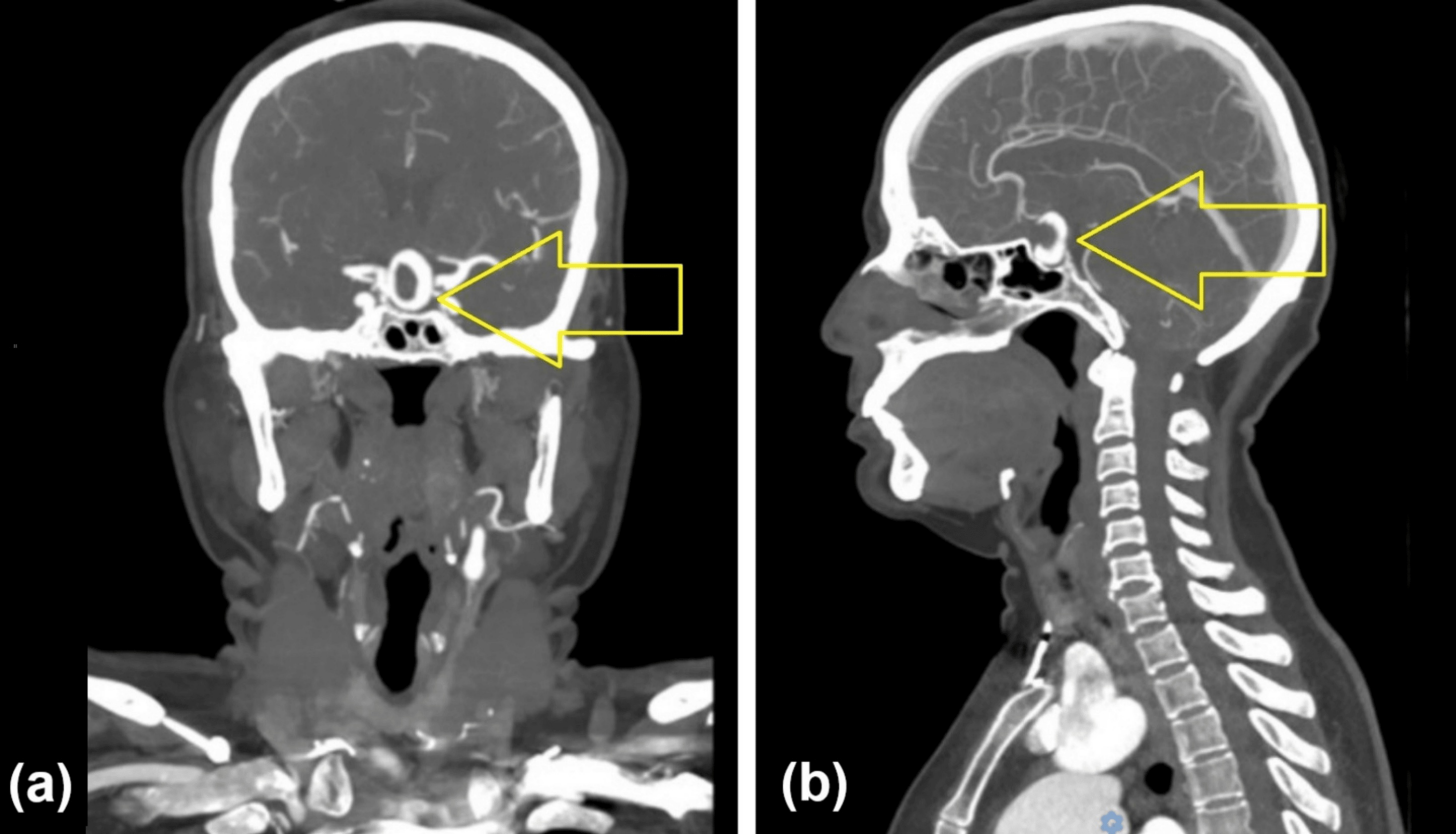
Computed tomography angiography. (A) Coronal image demonstrating a medially directed contrast-filled outpouching from the supraclinoid segment of the right internal carotid artery with a central non-enhancing thrombus (arrow). (B) Sagittal image demonstrating the aneurysmal sac with central thrombosis and suprasellar projection (arrow).

Computed tomography angiography of the head and neck then showed a medially directed contrast-filled outpouching from the supraclinoid segment of the right internal carotid artery with a central non-enhancing thrombus (Figure 4).

**Figure 5:**
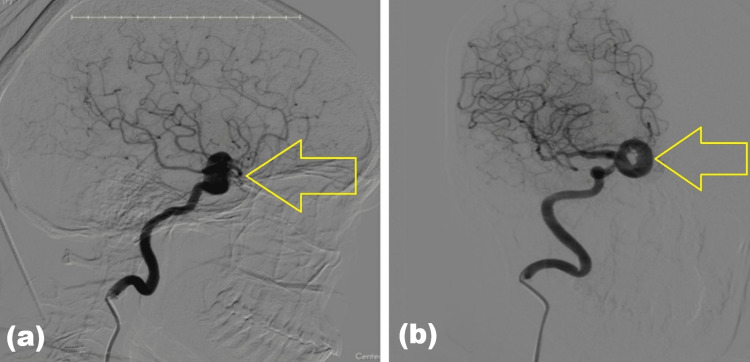
Digital subtraction angiography (DSA). (A) Lateral view demonstrating a saccular aneurysm arising from the supraclinoid segment of the right internal carotid artery (arrow). (B) Oblique view demonstrating a central filling defect consistent with partial thrombosis (doughnut configuration) (arrow).

Diagnostic digital subtraction angiography confirmed a medially directed aneurysm arising from the supraclinoid segment of the right internal carotid artery (superior hypophyseal aneurysm) with a central filling defect corresponding to partial thrombosis (Figure 5). The combined findings established the diagnosis of a rare doughnut-shaped partially thrombosed internal carotid artery aneurysm rather than a pituitary neoplasm.

**Figure 6:**
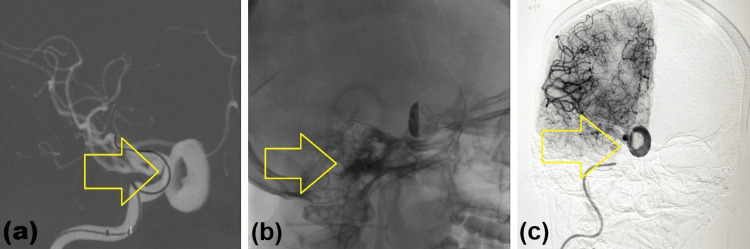
Endovascular treatment. (A) Microcatheter navigation across the aneurysm neck (arrow). (B) Deployment of the flow-diverter device across the internal carotid artery (arrow). (C) Post-deployment angiographic image demonstrating reduced intra-aneurysmal flow with contrast stasis (arrow).

The patient was planned for endovascular treatment after multidisciplinary discussion. Right common femoral arterial access was obtained, and vascular access was established with an 8-French catheter. A guiding catheter was advanced into the internal carotid artery, followed by placement of an intermediate catheter. A microwire and microcatheter system was navigated across the aneurysm neck under roadmap guidance. A flow-diverter device was then deployed across the diseased segment with excellent wall apposition. A 4.5 × 30 mm Silk Vista flow-diverting device (Balt, Montmorency, France), a self-expanding nitinol braided stent composed of 48 drawn filled tubing (DFT) wires with a platinum core for enhanced radiopacity, was deployed across the aneurysm neck. Given the close proximity of the ophthalmic artery to the deployment site, the potential risk of branch-vessel compromise from jailing was carefully considered. However, immediate post-deployment angiography demonstrated preserved ophthalmic artery flow along with significant intra-aneurysmal contrast stasis, consistent with effective flow diversion (Figure 6).

**Figure 7:**
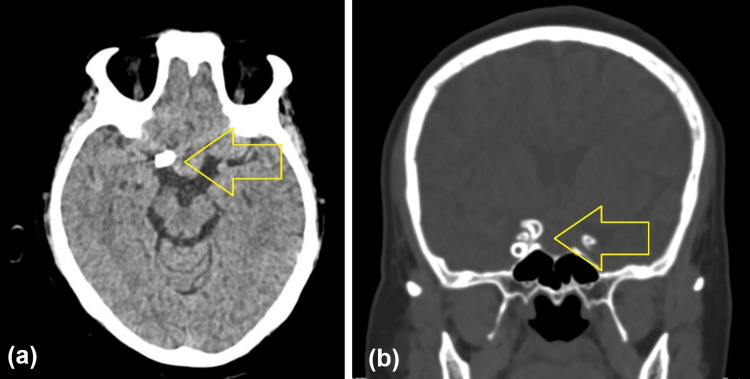
Post-treatment computed tomography (CT) of the brain. (A) Axial non-contrast CT demonstrating a hyperdense focus in the suprasellar region corresponding to the flow-diverter device, without hemorrhage (arrow). (B) Coronal non-contrast CT demonstrating the device in situ with no complications (arrow).

The vascular sheath was removed, and hemostasis was achieved by compression. No immediate procedural complication occurred. Figure 7 demonstrates post-procedural imaging.

**Figure 8:**
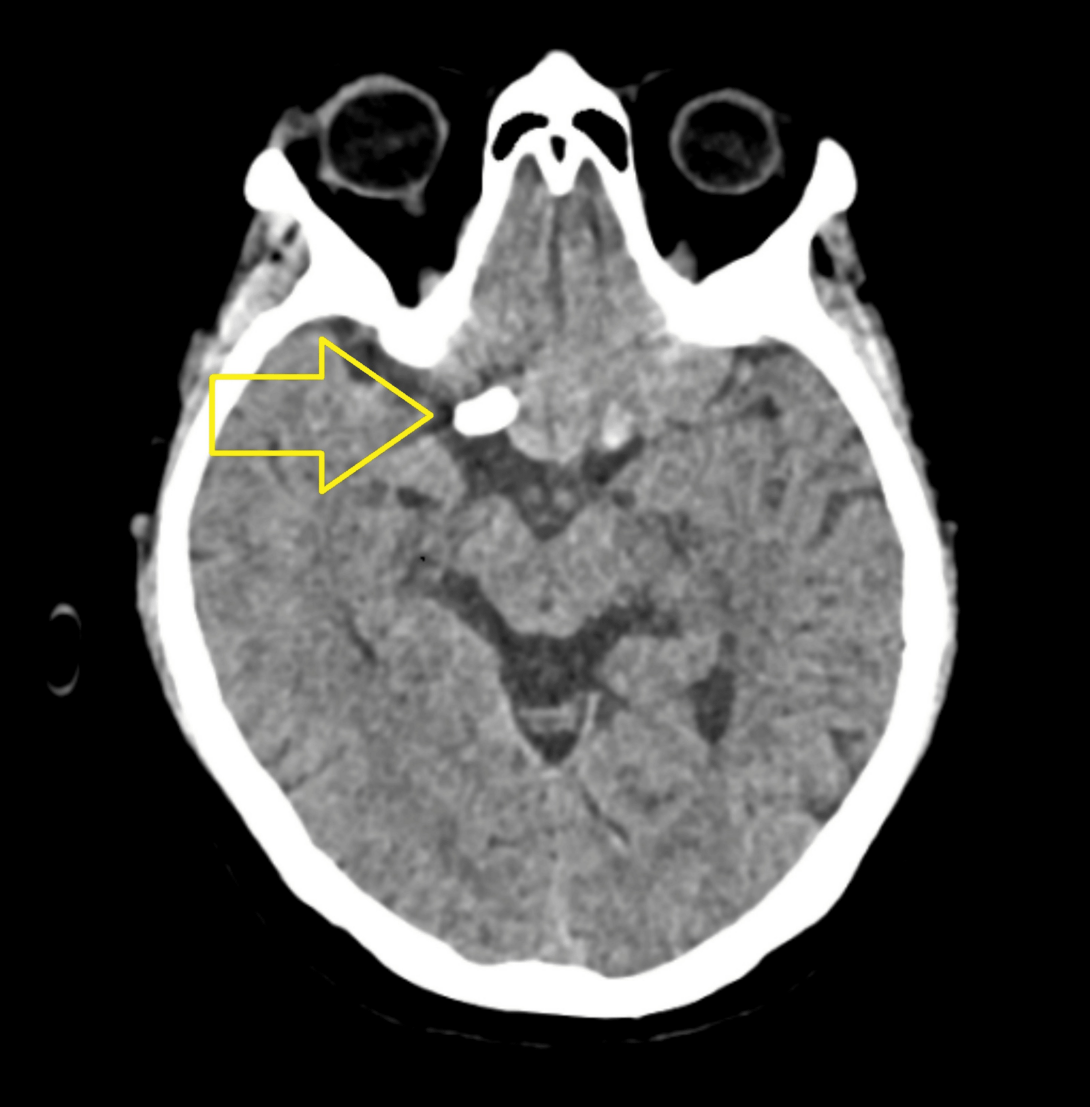
Axial non-contrast computed tomography (CT) of the brain at two-week follow-up demonstrating the flow-diverting device in situ in the suprasellar region, without evidence of complications (arrow).

The patient recovered well following the procedure and remained clinically stable, with no neurological deficits noted on follow-up. A non-contrast computed tomography of the brain performed at two weeks demonstrated the device in situ without any procedure-related complications (Figure 8). However, continued surveillance is recommended to monitor for potential delayed ischemic or visual complications.

## Discussion

This case illustrates an important diagnostic pitfall in skull base imaging. Aneurysms projecting into the sellar or suprasellar region may be interpreted as pituitary tumors when the patent lumen is small, and the thrombus burden is large. In the present patient, preserved vision, lack of endocrinological symptoms, and the relatively modest lesion size did not strongly suggest a vascular lesion. At the same time, the lesion’s relationship to the internal carotid arteries, its peripheral enhancement, and marked T2-weighted hypointensity were clues that the differential diagnosis should extend beyond adenoma and craniopharyngioma [1].

The magnetic resonance imaging appearance of partially thrombosed aneurysms varies according to thrombus age, organization, and residual luminal flow. In doughnut-shaped aneurysms, the residual lumen and thrombus geometry may create a ring-like configuration that becomes more apparent on contrast-enhanced studies or angiography. The pitfall is especially relevant in the sella because many clinicians instinctively favor pituitary macroadenoma when confronted with a small enhancing sellar-suprasellar mass. Non-contrast computed tomography and computed tomography angiography, therefore, remain decisive when a vascular mimic is suspected. In our case, hyperdensity on computed tomography supported the presence of thrombus rather than a simple cystic lesion, while computed tomography angiography and digital subtraction angiography clarified both diagnosis and treatment anatomy [2,3].

Modern endovascular management has made the treatment of ophthalmic-segment and paraophthalmic internal carotid artery aneurysms increasingly safe and effective. Long-term follow-up data from unruptured ophthalmic-segment aneurysms treated with flow-diverting devices have shown durable occlusion with acceptable visual outcomes, supporting their use in anatomically challenging lesions [5]. Studies of internal carotid artery aneurysms producing compressive neuro-ophthalmologic symptoms likewise suggest that complete occlusion and timely treatment are associated with better clinical recovery when mass effect has not become irreversible. Although our patient had normal vision preoperatively, the lesion’s close relationship to the optic apparatus made definitive exclusion important. No endocrine dysfunction was observed; however, sellar aneurysms may cause pituitary compression leading to hypofunction, and involvement of the stalk or posterior gland may disrupt antidiuretic hormone (ADH) transport, warranting endocrine evaluation [6,7].

Technical success in this case also reflects the broader maturation of flow-diverter therapy. Contemporary device series have reported high deployment success and progressive aneurysm occlusion across diverse anterior circulation aneurysms, including lesions unsuitable for simple coiling [8]. At the same time, partially thrombosed aneurysms remain biologically distinct from ordinary saccular aneurysms. Their thrombosed compartments may evolve over time, and altered hemodynamics can occasionally produce unexpected morphological changes, a phenomenon highlighted in recent reports on partially thrombosed aneurysms treated with flow-diversion strategies [9]. This means that angiographic follow-up remains essential even when the immediate post-procedural result is satisfactory.

The accumulating evidence base for flow diversion also supports its growing role in selected unruptured aneurysms, though recent systematic review data remind us that the quality of available comparative evidence is still imperfect [10]. In practical terms, individual lesion morphology, branch vessel anatomy, thrombus burden, and local technical expertise continue to matter greatly in treatment selection. Finally, although anatomically unrelated to the present lesion, recent reporting on rare vascular anomalies in other body regions has similarly emphasized the value of multimodal imaging, staged treatment, and multidisciplinary decision-making when uncommon vascular lesions present as diagnostic surprises [11,12].

## Conclusions

This case describes a rare doughnut-shaped partially thrombosed aneurysm arising from the ophthalmic segment of the right internal carotid artery that closely mimicked a pituitary macroadenoma on initial magnetic resonance imaging. The diagnosis was corrected only because atypical imaging clues prompted further vascular studies before surgery was attempted. That sequence was critical, since a routine transsphenoidal approach to an unrecognized aneurysm could have been catastrophic. The case, therefore, highlights the importance of recognizing vascular red flags in sellar and suprasellar masses, particularly hyperdensity on computed tomography, marked T2-weighted hypointensity, peripheral rim enhancement, and close internal carotid artery relationship. It also demonstrates the value of endovascular flow-diverter treatment in achieving safe parent-vessel reconstruction and early exclusion of the aneurysm from the circulation. Greater awareness of this rare radiologic masquerader among emergency physicians, radiologists, neurosurgeons, and neurointerventionists can improve diagnostic accuracy, prevent inappropriate surgery, and lead to better patient outcomes.
